# PIP-EL: A New Ensemble Learning Method for Improved Proinflammatory Peptide Predictions

**DOI:** 10.3389/fimmu.2018.01783

**Published:** 2018-07-31

**Authors:** Balachandran Manavalan, Tae Hwan Shin, Myeong Ok Kim, Gwang Lee

**Affiliations:** ^1^Department of Physiology, Ajou University School of Medicine, Suwon, South Korea; ^2^Institute of Molecular Science and Technology, Ajou University, Suwon, South Korea; ^3^Division of Life Science and Applied Life Science (BK21 Plus), College of Natural Sciences, Gyeongsang National University, Jinju, South Korea

**Keywords:** proinflammatory peptide, ensemble learning, random forest, machine learning, immunotherapy

## Abstract

Proinflammatory cytokines have the capacity to increase inflammatory reaction and play a central role in first line of defence against invading pathogens. Proinflammatory inducing peptides (PIPs) have been used as an antineoplastic agent, an antibacterial agent and a vaccine in immunization therapies. Due to the advancement in sequence technologies that resulted an avalanche of protein sequence data. Therefore, it is necessary to develop an automated computational method to enable fast and accurate identification of novel PIPs within the vast number of candidate proteins and peptides. To address this, we proposed a new predictor, PIP-EL, for predicting PIPs using the strategy of ensemble learning (EL). Our benchmarking dataset is imbalanced. Thus, we applied a random under-sampling technique to generate 10 balanced models for each composition. Technically, PIP-EL is the fusion of 50 independent random forest (RF) models, where each of the five different compositions, including amino acid, dipeptide, composition–transition–distribution, physicochemical properties, and amino acid index contains 10 RF models. PIP-EL achieves the Matthews’ correlation coefficient (MCC) of 0.435 in a 5-fold cross-validation test, which is ~2–5% higher than that of the individual classifiers and hybrid feature-based classifier. Furthermore, we evaluate the performance of PIP-EL on the independent dataset, showing that our method outperforms the existing method and two different machine learning methods developed in this study, with an MCC of 0.454. These results indicate that PIP-EL will be a useful tool for predicting PIPs and for researchers working in the field of peptide therapeutics and immunotherapy. The user-friendly web server, PIP-EL, is freely accessible.^1^

## Introduction

Inflammation is modulated by a host of molecular regulators, such as cytokines, complement eicosanoids, growth factors, and peptides (1). The key modulators of inflammation are cytokines, which participate in both acute and chronic inflammation. Cytokines can be classified based on the nature of immune response, cell type, location, and receptor type, used for signalling. Critical proinflammatory cytokines include interleukin (IL)-1, IL-6, IL-8, IL-12, IL-18, interferon (IFN)-γ, and tumour necrosis factor (TNF)-α (2, 3).

Peptides are gaining momentum in pharmaceutical research and development because of their improved selectivity, high efficacy, tolerability, and biosafety. More than 150 peptide therapeutics are currently being evaluated in clinical trials (4). Besides their attractive pharmacological profile and intrinsic properties, peptides provide an excellent starting point for novel therapeutics. The role of peptides in inflammation can be proven *via* pathophysiological events, such as the release of tachykinins from sensory nerves for mediation of neurogenic inflammation and bradykinin from local and systemic inflammation (5). Peptides that induce proinflammatory cytokines are known as proinflammatory inducing peptides (PIPs), which can be utilized as therapeutic candidates to alleviate and cure various diseases (6, 7). For example, *Helicobacter pylori* produces a cecropin-like peptide [i.e., Hp(2–20)] that induces a proinflammatory response in human neutrophils, thereby acting as a potent antineoplastic agent (8). Prostate-specific antigen peptides have also been used in immunotherapies (9). Human cathelicidin LL-37 proinflammatory peptide has a role in the pathogenesis of rheumatoid disease, atherosclerosis, and antibacterial activities (10, 11). The gG-2p20 peptide induces a proinflammatory response by recruiting and activating phagocytic cells, thus reducing the function of NK cells (12).

Identification of PIPs is one of the hot topics in immunoinformatics and computational biology. An increasing number of PIPs have been experimentally identified and validated (13), it is expected that the number of PIPs will grow rapidly. As for the discovery of PIPs from protein primary sequence, experimental methods are time consuming, expensive, and difficult to be applied in a high-throughput manner. Therefore, development of a sequence-based computational method is needed to identify the possible potential candidates prior to the experimental procedure. To this end, several computational studies have focused on the prediction of different types of immune epitopes, including IL-4-inducing peptides (14), IL-10-inducing peptides (15), anti-inflammatory cytokine-inducing peptides (16), MHC binders (17), T-cell epitopes (17, 18), B-cell epitopes (19), and allergenicity (20–22). However, a few methods focused on predicting specific proinflammatory cytokine (i.e., IL17 and IFN-γ) inducing peptides (7, 23). Only one method (i.e., ProInflam) is available to predict general proinflammatory responses (i.e., IL1α, IL1β, TNFα, IL12, IL18, and IL23) that induce peptides (6).

Although ProInflam has contributed to stimulating development in this area, more work is needed for the following reasons. (i) With the rapidly increasing number of pro-inflammatory inducing epitopes in the Immune Epitope Database (IEDB), it remains an important and urgent task to develop more accurate prediction methods with a larger benchmarking dataset. (ii) The feature space used by the existing method is incomplete. Thus, additional potent features are needed for characterization. Owing to these deficiencies, other methods are necessary to accurately predict PIPs by taking advantage of machine learning (ML) algorithms, based on high-quality benchmarking datasets.

**Figure 1:**
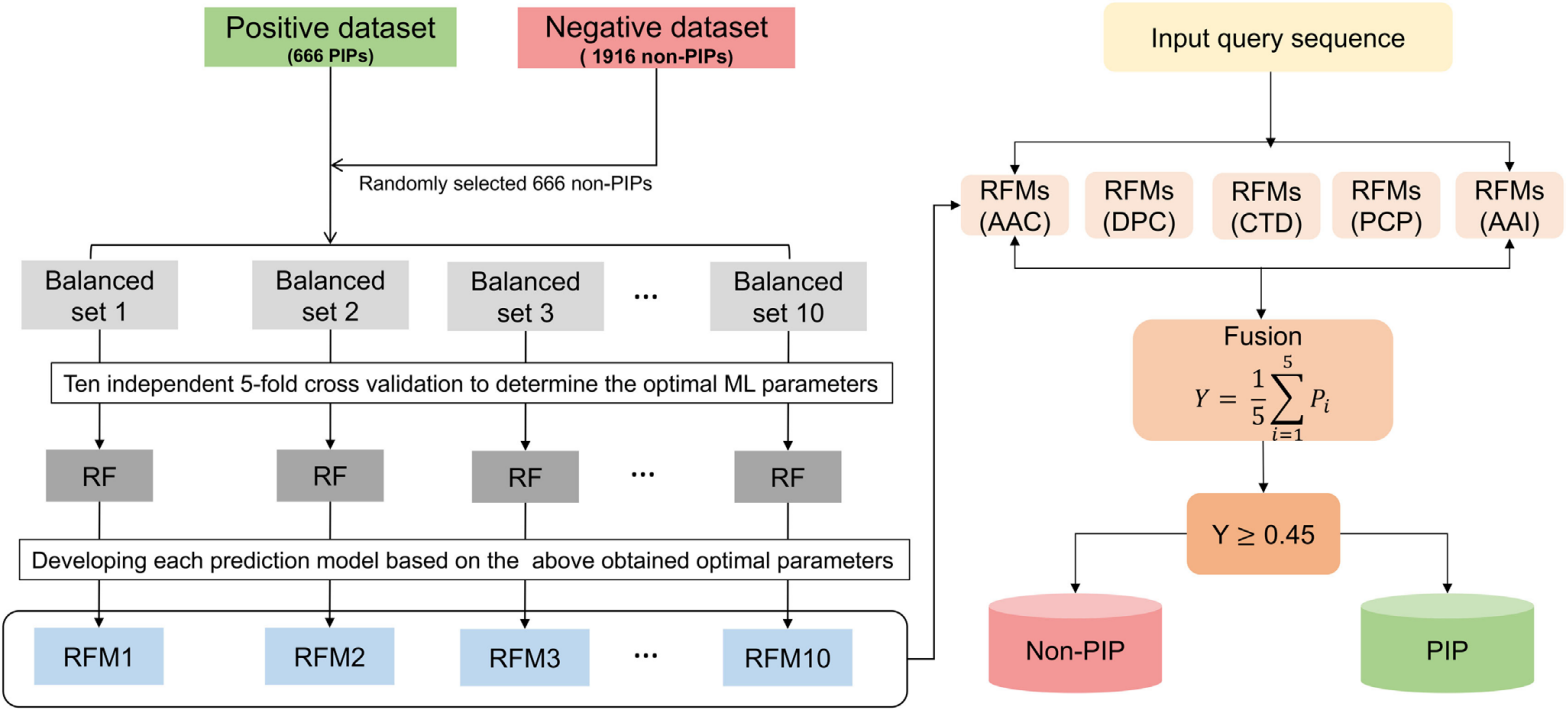
Overall framework of PIP-EL. The random under-sampling technique was applied to handle the imbalanced benchmarking dataset, generating 10 balanced RFMs. In total, 50 prediction models (i.e., 10 RFMs × 5 compositions = 50 RFMs) were obtained for five different compositions, including amino acid composition, dipeptide composition, composition–transition–distribution, amino acid index, and physicochemical properties. All these models were utilized to generate the final ensemble model.

In this study, we constructed a nonredundant (nr) dataset of experimentally validated PIPs and non-PIPs extracted from the IEDB, sharing relatively low sequence similarities (i.e., no more than 80%) to avoid performance bias. From the nr dataset, we randomly select 80% of the data as the benchmarking dataset and 20% as the independent dataset. Various features extracted from the benchmarking dataset, including amino acid composition (AAC), dipeptide composition (DPC), composition–transition–distribution (CTD), amino acid index (AAI), and physicochemical properties (PCP), an input to the random forest (RF) algorithm to develop classification models. Because our benchmarking dataset is imbalanced, we applied a random under-sampling technique and generated 10 balanced models for each composition. Technically, PIP-EL is the fusion of 50 models from five different compositions (Figure 1). In addition to PIP-EL, we also develop extremely randomized trees (ERT) and support vector machine (SVM) methods using the same protocol as PIP-EL. Note that when objectively evaluated using an independent dataset, PIP-EL displays superior performance compared to the currently available method (i.e., ProInflam) and two other methods (i.e., ERT and SVM) developed in this study.

## Materials and Methods

### Dataset Construction

To build an ML model, a well-curated and clear-cut dataset is required. Therefore, we extracted experimentally validated positive (i.e., 1,502 PIPs) and negative (i.e., 3,335 non-PIPs) linear peptides or epitopes from the IEDB (13, 24, 25). A peptide was considered positive if it induced any one of the proinflammatory cytokines (i.e., IL1α, IL1β, TNFα, IL6, IL8, IL12, IL17, IL18, and IL23) in T-cell assays of human and mouse. Similarly, linear peptides tested negative in inducing proinflammatory cytokines were considered negative. Due to their lower frequency, we excluded the peptides that have a length lower than 5 or greater than 25 amino acid residues from our dataset, since such inclusions may form an outlier during prediction model development. To generate an nr dataset, we eliminate redundant peptides using CD-HIT by applying a sequence identity threshold of 0.8, indicating that sequence identity between any two sequences greater than 80% is discarded. Using a more stringent criterion, such as 30 or 40%, as imposed in Ref. (19, 26, 27), could improve the credibility reliable of the model. However, in this study, we do not use such a stringent criterion, because our currently available data does not allow it. Otherwise, the number of samples for some subsets would be insufficient for showing statistical significance.

Finally, we obtained an nr dataset of 833 PIPs and 2,395 non-PIPs, whose size is ~4-fold bigger than the dataset used in the previous method (i.e., ProInflam). Our dataset contained nine proinflammatory cytokines, including six of them (i.e., IL1α, IL1β, TNFα, IL12, IL18, and IL23) used by ProInflam. From this nr dataset, 80% of the data was randomly selected as the benchmarking dataset (i.e., 666 PIPs and 1,916 non-PIPs) to develop a prediction model, whereas the remaining 20% was considered the independent dataset (i.e., 167 PIPs and 479 non-PIPs).

### Input Features

For the computational approach, each peptide sequence is represented as a numerical vector (i.e., features) input to ML algorithms for binary classification (i.e., PIP or non-PIP). Here, we used five different compositions, as follows.

#### Amino Acid Composition

Amino acid composition is the percentage of natural amino acids in a given peptide sequence, having a fixed length of 20 features. It was calculated using the following equation:

(1) $$\text{AAC(}i\text{)}=\frac{\text{Frequency of amino acid }(i)}{\text{Peptide length}},$$

where *i* can be one of 20 possible amino acids.

#### Dipeptide Composition

Dipeptide composition represents the frequency of dipeptides normalized by all possible dipeptide combinations, having a fixed length of 400 features. It is calculated as follows:

(2) $$\text{DPC(}i\text{)}=\frac{\text{Frequency of dipeptide }(i)}{\text{Total number of all possible dipeptides}},$$

where *i* can be one of 400 possible dipeptides.

#### Composition–Transition–Distribution

The CTD feature was introduced by Dubchak et al. (28) for predicting protein-folding classes. Thereafter, it was successfully applied in various sequence-based classification algorithms (29–33). CTD represents the distribution of amino acid patterns along the primary sequence, based on their physicochemical or structural properties. There are seven physiochemical properties, including hydrophobicity, polarizability, normalized van der Waals volume, secondary structure, polarity, charge, and solvent accessibility.

All amino acids are divided into three groups: polar, neutral, and hydrophobic. C consists of three percentage composition values for a given peptide: polar, neutral, and hydrophobic. T consists of the percentage frequency of a polar followed by a neutral residue or of a neutral by a polar residue. It may also consist of a polar, followed by a hydrophobic residue or a hydrophobic followed by a polar residue. It may also consist of a neutral, followed by a hydrophobic or a hydrophobic, followed by a neutral residue. D consists of five values for each of the three groups. It measures the chain length, within which the first, 25, 50, 75, and 100% of the amino acids of a specific property are located. There are three descriptors and 3(C) + 3(T) + 5 × 3(D) = 21 descriptor values for a single amino acid attribute. Consequently, seven different amino acid attributes produce a total of 7 × 21 = 147 features.

#### AAI-Based Features

The AAIndex database contains amino acid indices of various physicochemical and biochemical properties (34). Saha et al. classified these amino acid indices into eight clusters, and the central indices of each cluster were named as high-quality amino acid indices (35): BLAM930101, BIOV880101, MAXF760101, TSAJ990101, NAKH920108, CEDJ970104, LIFS790101, and MIYS990104. We utilize this information, which encodes as a 160 (20 × 8 = 160)−dimensional vectors from the peptide sequence.

Additionally, we averaged eight high-quality amino acid indices (i.e., a 20-dimensional vector) as an input feature. Our preliminary analysis showed that these two feature sets (i.e., 160 and 20) produce similar results. Thus, we use the 20-dimensional vector as the final one to save computational time.

#### PCP-Based Features

Frequencies of the following features are directly computed from the sequence consisting of: (1) hydrophobic (i.e., F, I, W, L, V, M, Y, C, and A); (2) hydrophilic (i.e., R, K, N, D, E, and P); (3) neutral (i.e., T, H, G, S, and Q); (4) positively charged (i.e., K, H, and R); (5) negative-charged (i.e., D and E); (6) turn-forming residues fraction [i.e., (N + G + P + S)/*n*, where *n* = sequence length]; (7) absolute charge per residue (i.e., $\frac{\text{R}+\text{K}-\text{D}-\text{E}}{n}-0.03$); (8) molecular weight; and (9) aliphatic index [i.e., (A + 2.9V + 3.9I + 3.9L)/*n*].

All of the above feature vectors are normalized in the range of 0–1, according to the formula described in our previous study (36).

### ML Algorithms

In this study, three ensemble models are proposed using three different ML algorithms, including RF (37), SVM (38), and ERT (39), implemented per the Scikit-Learn package (v0.18) (40). A brief description of each algorithm and how it is used in this study follows.

#### Support Vector Machine

Support vector machine is used to develop both classification and regression models based on the principle of structural risk minimization, which has been successfully applied in many bioinformatics fields (41–44). SVM maps the input features into a high-dimensional feature space and then determines the optimal separating hyperplane between two classes. In our study, a Gaussian radial-basis function (RBF) is used to obtain the classification hyperplane. An RBF-SVM requires the optimization of two critical parameters*: C* and γ*. C* controls the trade-off between correct classification and a large margin and γ controls how fast RBF similarity vanishes with growing Euclidean distance between vectors. Therefore, a grid search is conducted in the following ranges: *C* from 2^−15^ to 2^10^ and γ from 2^−10^ to 2^10^ in log_2_-scale, conducted to tune the SVM parameters (i.e., *C* and γ).

#### Random Forest

Random forest is one of the most successful ML method that utilizes hundreds or thousands of independent decision trees to perform classification and regression (37), which has been widely used in bioinformatics (36, 45, 46). RF combined the concept of bagging and random feature selection. For a given training data set (*D*), generate a new training data set (*D_i_*) by drawing N bootstrapped samples from *D* uniformly and with replacement, which is called as bootstrap sample. Grow a tree using *D_i_* repeat the following steps at each node of the tree until its fully grown (i) select *mtry* random features from the original features and select the best variable by optimizing the impurity criteria, and (ii) split the node into two child nodes. The tree grows until the number of data in the node smaller than the given threshold (*nsplit*). Repeating the above-mentioned steps to build a large number (*ntree*) of classification trees. To classify a test data, input features are passed through from the root to end node of each tree based on the predetermined splits. The majority of the class from the forest is considered as the final one. The three most influential parameters are *ntree, mtry*, and *nsplit*. A grid search range is given in Table S1 in Supplementary Material, optimized using a 5-fold cross-validation.

#### Extremely Randomized Trees

Extremely randomized trees belong to another class of ensemble methods widely used for developing classification and regression models (39). ERT aim to further reduce the variance of the prediction model by adding stronger randomization technique. The ERT algorithm is similar to the RF method. Specifically, ERT uses the whole dataset instead of bootstrap sample used in RF, but the trees are generated randomly. The random selection at each node reduces the tree construction time as fewer tests are performed to search for the best split. Furthermore, the parameter optimization procedure in ERT is the same as that in the RF method.

### Performance Evaluation

Predictions were classified into four groups: true positive (TP) is the number of PIPs correctly predicted as PIPs; true negative is the number of non-PIPs correctly predicted as non-PIPs; false positive (FP) is the number of non-PIPs wrongly predicted as PIPs; and false negative is the number of PIPs wrongly predicted as non-PIPs. To measure prediction quality, we used the following five metrics: sensitivity, specificity, accuracy, the Matthews’ correlation coefficient (MCC), and the area under receiver operating characteristics (ROC) curve. All these metrics are commonly used in the literature to measure the binary classification (47–50):

(3) $$\{\begin{array}{c} \text{Sensitivity}=\frac{\text{TP}}{\text{TP}+\text{FN}}\\ \text{Specificity}=\frac{\text{TN}}{\text{TN}+\text{FP}}\\ \text{Accuracy}=\frac{\text{TP}+\text{TN}}{\text{TP}+\text{TN}+\text{FP}+\text{FN}}\\ \text{MCC}=\frac{\text{TP}\times \text{TN}-\text{FP}\times \text{FN}}{\sqrt{\text{(TP}+\text{FP)(TP}+\text{FN)(TN}+\text{FP)(TN}+\text{FN)}}}, \end{array}$$

AUC is the area under the ROC curve, representing the relationship between TP rate and FP rate of the model. The AUC is an indicator of the performance quality of the binary classifier. The AUC value of 0.5 is equivalent to random prediction, but, an AUC value of 1 represents perfection.

### Cross-Validation

There are three kinds of cross-validations (CVs): *k*-fold CV, jackknife CV, and independent dataset (51) are often used to evaluate the anticipated success rate of a predictor. Among these three approaches, jackknife test is deemed the least arbitrary and most objective one as demonstrated by Eqs 28–32 of Ref. (52), and hence has been widely used in bioinformatics because it could produce unique outcome (43, 53–62). However, it is time- and source-consuming. Thus, in this paper, we used 5-fold CV to examine the proposed models, where benchmarking data set is randomly divided into five parts, from which four parts were used for training, and the fifth part was used for testing. This process was repeated until all the parts were used at least once as a test set, and the overall performance with all five parts was evaluated.

## Results

### Compositional and Positional Information Analysis

**Figure 2:**
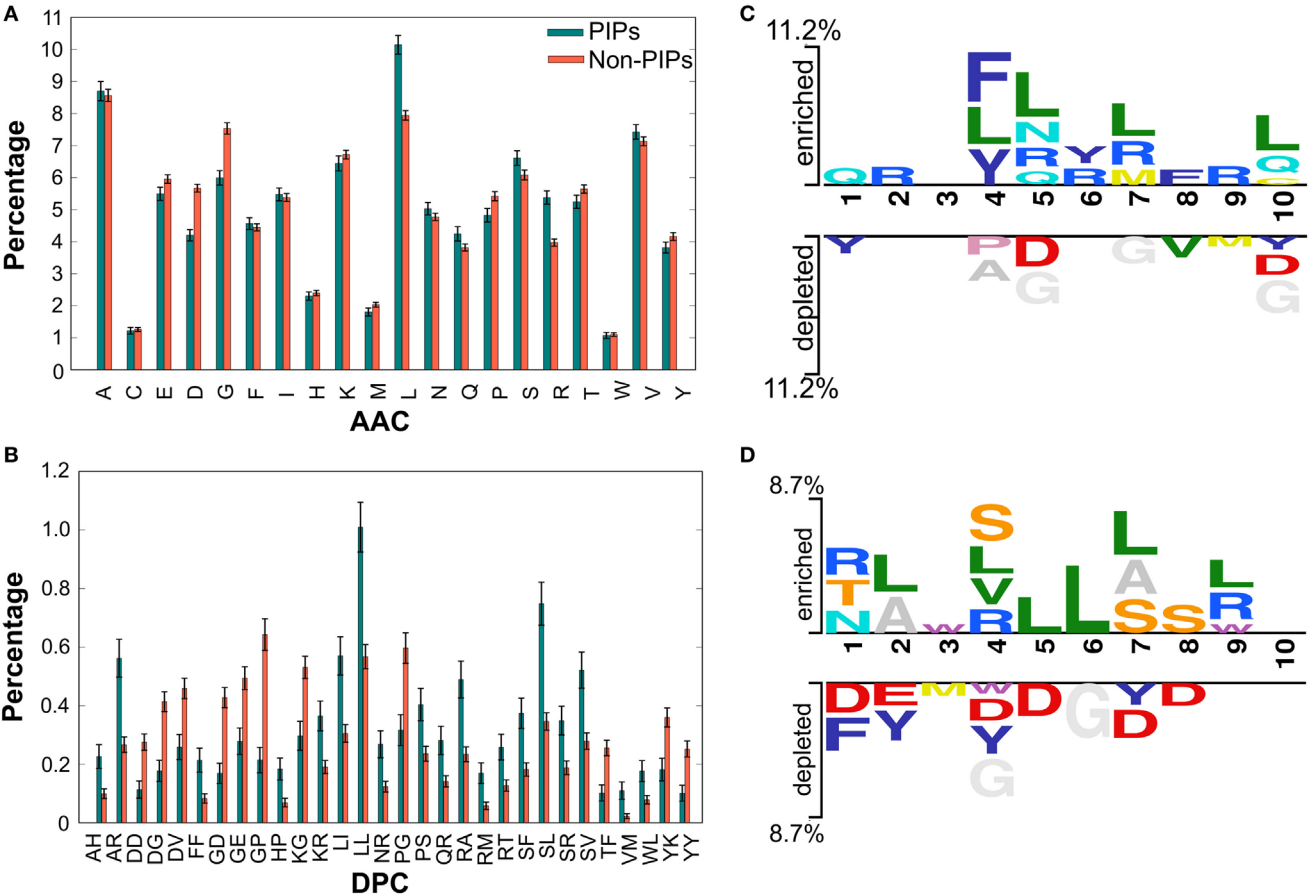
Compositional and positional preference analysis. **(A,B)** Respectively represent the amino acid and dipeptide preferences between proinflammatory inducing peptides (PIPs) and non-PIPs. In **(B)** significantly different top-30 dipeptides are shown. **(C,D)** Represent positional conservation of 10 residues at N- and C-terminal between PIPs and non-PIPs, respectively, generated using two sample logos.

We performed compositional analysis using the combined dataset (i.e., benchmarking and independent). AAC analysis revealed that average composition of certain residues, including Arg and Leu, were dominant in PIPs. However, Gly, Asp, and Pro were dominant in non-PIPs (Welch’s *t*-test; *P* ≤ 0.01) (Figure 2A). Furthermore, DPC analysis revealed that 21% of dipeptides differed significantly between PIPs and non-PIPs (Welch’s *t*-test; *P* ≤ 0.01). Of these, the top-10 most abundant dipeptides in PIPs and non-PIPs were FF, SL, SR, SF, SV, LL, LI, RT, RA, and RM and GP, GE, GD, YK, YY, KG, DG, DD, DV, and PG, respectively (Figure 2B). These results suggest that the most abundant dipeptides in PIPs consist primarily of pairs of aliphatic–aliphatic, positively charged-aliphatic, and hydroxyl group-aliphatic or -aromatic amino acids. However, the most abundant dipeptides in the non-PIPs were negatively charged-negatively charged, small-positive or -negatively charged, and aromatic-aromatic or -positively charged amino acids. Overall, significant differences observed in compositional analysis could be integrated into ML algorithms to improve prediction performances. Thus, we considered them as input features.

To understand the positional information of each residue, a sequence logo of the first 10 residues from the N- and the C-terminal of PIPs and non-PIPs were generated using two sample logos.^2^ To test their statistical significance, the height of the peptide logos were scaled (*t*-test by *P* < 0.05). At the N-terminal, we found that, compared to other amino acids, R, at positions 2, 5, 6, 7, and 9; L, at positions 4, 5, 7, and 10; and Q, at positions 1, 5, and 10 were significantly overrepresented. Alternatively, negatively charged residue D, at positions 5 and 10 and G, at positions 5, 7, and 10 were significantly underrepresented (Figure 2C). No significant amino acids were found at enriched position 3 or the depleted positions 2, 3, and 6. C-terminal R, at positions 1, 4, and 9; L, at positions 2, 4, 5, 6, 7, and 9; and S/T, at positions 1, 4, 7, and 8 were significantly overrepresented. Alternatively, negatively charged residues D/E, at positions 1, 2, 4, 5, 7, and 8 and Y, at positions 2, 4, and 7 were significantly underrepresented (Figure 2D). No significant amino acids were found at enriched position 10 or the depleted positions 9 and 10. These results suggest that comparatively residues, R and L, are preferred in PIPs. This is consistent with the AAC analysis observation. Furthermore, positional preference analysis will be helpful for experimenters who design *de novo* PIPs and substitute amino acids at particular positions to make the peptides more effective.

### Construction of PIP-EL

We employed the RF method to construct an ensemble predictor, called PIP-EL. A framework for the construction of PIP-EL is shown in Figure 1. Note that our benchmarking dataset was imbalanced (i.e., 666 PIPs and 1,916 non-PIPs). Thus, it needed special treatment while developing the prediction models. Although several solutions for the imbalanced problem has been proposed in the literature (63, 64), we considered the most straightforward random under-sampling technique, where the majority class was subjected to random sampling, that was equal to the minority class in each subset. Here, we generated 10 different balanced datasets (i.e., B1–10) with the ratio of 1:1, or 663 PIPs:663 non-PIPs, randomly selected from the original. This step ensured that each sample from the majority class was used at least once. For a given feature set (e.g., AAC), we carried out a 5-fold CV grid search to optimize parameters (see Table S1 in Supplementary Material). However, other hyper-parameters remained at their default value. Considering that one-time 5-fold CV with random portioning might produce biased ML parameters, we repeated 5-fold CV 10 more times and considered median ML parameters as the optimized value. This was utilized to develop a final prediction model. This CV procedure applied to B1–10 and resulted in 10 models (i.e., RF1-10) for each composition.

Ensemble learning can be formed by fusing an array of independent models *via* voting or averaging the outcome of independent predictions. Whereas this approach is computationally expensive, it has been shown to produce more accurate and robust results than constituent models. This approach has been successfully applied in various bioinformatics applications (65–68). In this study, we generated an ensemble predictor for PIPs, as follows:

(4) $$\begin{array}{l} {\text{RF}}^{\text{E}}=\text{RFMs}(\text{AAC})\forall \text{RFMs}(\text{DPC})\\ \;\forall \text{ RFMs}(\text{PCP})\forall \text{RFMs(CTD)}\\ \;\forall \text{ RFMs}(\text{AAI}). \end{array}$$

RFM refers to the RF model. The ensemble predictor, RF^E^, contained 5 composition-based × 10 balanced models = 50 models. ∀ denotes the fusing operator. After fusion, we optimized the average probability cut-off value with respect to MCC using grid search to define the class (PIPs or non-PIPs). The cut-off of 0.45 produced the best performance, hence we fixed this as an optimal cut-off value. Thus,

(5) $$\text{D}\in \{\begin{array}{c} \text{PIP, if }Y\geq 0.45,\\ \text{non-PIP, otherwise}. \end{array}$$

Finally, PIP-EL was composed of 50 prediction models, and each classifier used their own optimal parameters.

### Comparison of PIP-EL With Individual Composition-Based Classifiers

**Figure 3:**
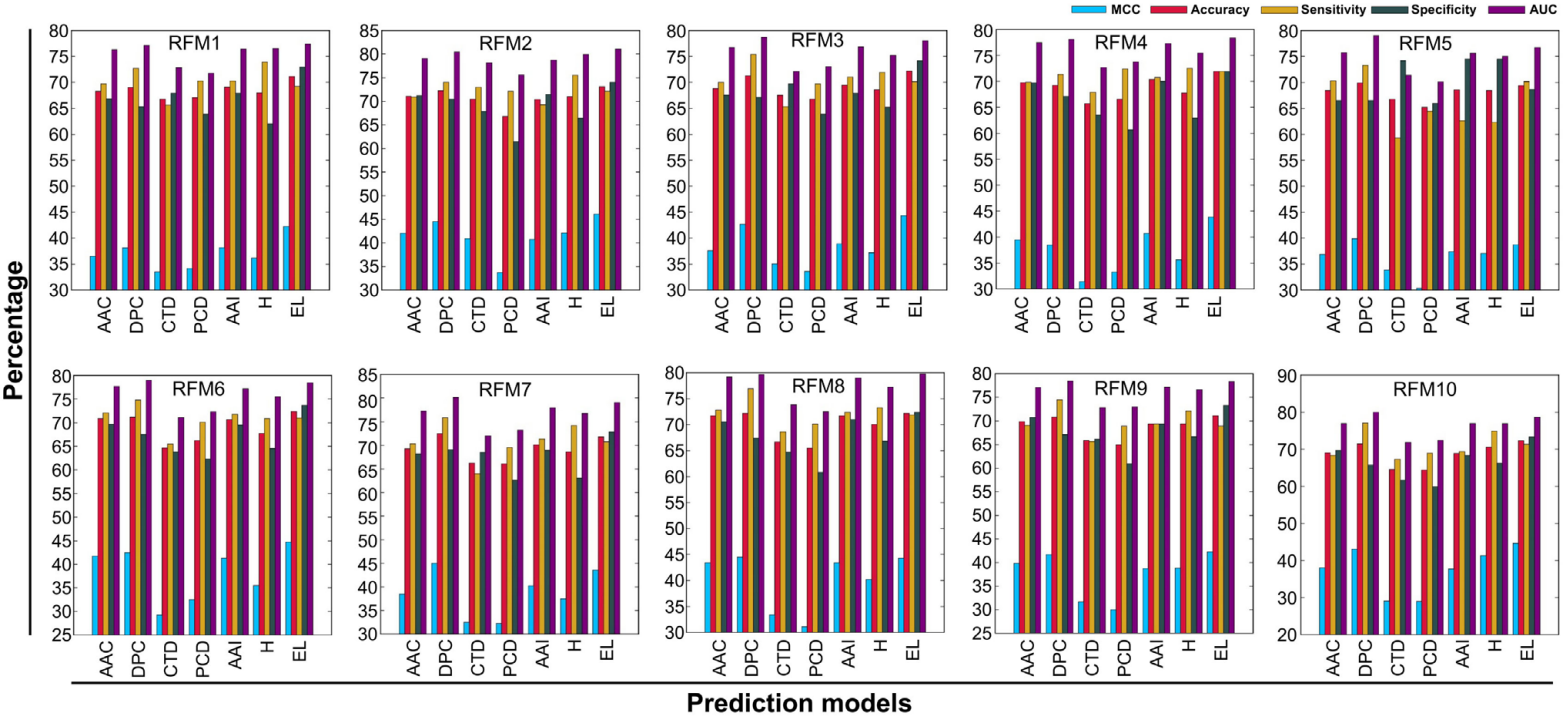
Performance of five composition-based models, hybrid model, and ensemble model on 10 alternatively balanced datasets. *X*- and *Y*-axes, respectively, correspond to prediction models and performance (%). RFMX (i.e., *X* = 1–10) represents RFM from balanced dataset *X*.

In addition to PIP-EL, we also developed five different ensemble models by fusing various combinations of five composition-based models. PIP-EL produced the best performance among them (data not shown). Thus, we considered it as the final model. To demonstrate the performance of PIP-EL, we compared it with five composition-based models (i.e., AAC, DPC, CTD, PCP, and AAI) and the hybrid classifier (i.e., H: combination of five composition as the input feature to the RF) with the benchmarking dataset. Figure 3 shows that PIP-EL performed consistently better than other models, both in terms of MCC and accuracy. The average values of these metrics, with SD, are shown in Table 1, showing that PIP-EL achieved values of 0.435 and 0.717 for MCC and accuracy, respectively. Indeed, the corresponding metrics were ~2–11 and ~1–6% higher than those achieved by individual compositions, indicating superiority of PIP-EL. According to *P*-value <0.05, PIP-EL performed better than AAC, DPC, AAI, and H. It performed significantly better than CTD- and PCP-based models. Moreover, PIP-EL has an advantage over other composition-based models because it covers various angles of sequence information.

**Table 1:** Performance comparison of random forest (RF)-based ensemble method with RF-based other classifiers on benchmarking dataset.

Features	Matthews’ correlation coefficient (MCC)	Accuracy	Sensitivity	Specificity	AUC	*P*-value
Amino acid composition (AAC)	0.394 ± 0.022	0.697 ± 0.011	0.703 ± 0.013	0.691 ± 0.016	0.769 ± 0.009	0.288
Dipeptide composition (DPC)	0.420 ± 0.023	0.709 ± 0.012	0.746 ± 0.017	0.673 ± 0.014	0.780 ± 0.011	0.651
Composition–transition–distribution (CTD)	0.330 ± 0.032	0.665 ± 0.016	0.662 ± 0.033	0.668 ± 0.034	0.729 ± 0.019	**0.001**
Physicochemical properties (PCP)	0.320 ± 0.017	0.659 ± 0.008	0.696 ± 0.020	0.622 ± 0.017	0.725 ± 0.013	**0.0006**
Amino acid index (AAI)	0.397 ± 0.018	0.698 ± 0.009	0.698 ± 0.026	0.699 ± 0.019	0.772 ± 0.010	0.370
Hybrid	0.381 ± 0.022	0.690 ± 0.011	0.721 ± 0.036	0.658 ± 0.033	0.762 ± 0.014	0.149
PIP-EL	0.435 ± 0.019	0.717 ± 0.010	0.707 ± 0.010	0.727 ± 0.015	0.788 ± 0.011	–

*The first column corresponds to the performance of individual feature group, hybrid feature, and ensemble learning. The column 2–6 respectively represent the MCC, accuracy, sensitivity, specificity, and AUC value, where each value shown as the average ± SD of 10 alternative balanced datasets. The last column represents a pairwise comparison of AUC between PIP-EL and the other methods using a two-tailed t-test. P ≤ 0.05 indicates a statistically meaningful difference between PIP-EL and the selected composition (shown in bold)*.

### Comparison of PIP-EL With Other ML-Based Methods

Generally, it is quite difficult to choose a suitable ML method for a given problem because of the problem-specific nature of the ML algorithms. Hence, it is essential to explore the performance of different ML methods while using the same benchmarking dataset and selecting the best one, instead of selecting method arbitrarily. In addition to PIP-EL, we developed an ensemble model using ERT and SVM. Here, the procedure of ML parameter optimization for the other two methods and the construction of ensemble models were the same as PIP-EL. Surprisingly, ERT and SVM exhibited their best performances using the ensemble model (Tables 2 and 3) when compared to their individual composition and hybrid models.

**Table 2:** Performance comparison of extremely randomized trees (ERT)-based ensemble method with ERT-based other classifiers on benchmarking dataset.

Features	Matthews’ correlation coefficient (MCC)	Accuracy	Sensitivity	Specificity	AUC	*P*-value
Amino acid composition (AAC)	0.367 ± 0.025	0.694 ± 0.034	0.612 ± 0.108	0.743 ± 0.074	0.752 ± 0.015	0.09
Dipeptide composition (DPC)	0.375 ± 0.022	0.686 ± 0.011	0.636 ± 0.022	0.737 ± 0.017	0.757 ± 0.013	0.325
Composition–transition–distribution (CTD)	0.295 ± 0.030	0.647 ± 0.015	0.607 ± 0.019	0.687 ± 0.018	0.694 ± 0.017	**0.00002**
Physicochemical properties (PCP)	0.313 ± 0.030	0.656 ± 0.015	0.632 ± 0.018	0.680 ± 0.017	0.705 ± 0.013	**0.0002**
Amino acid index (AAI)	0.371 ± 0.028	0.685 ± 0.014	0.648 ± 0.015	0.722 ± 0.018	0.748 ± 0.013	0.143
Hybrid	0.348 ± 0.022	0.674 ± 0.007	0.645 ± 0.016	0.703 ± 0.006	0.733 ± 0.012	**0.02**
Ensemble learning (EL)	0.423 ± 0.024	0.712 ± 0.012	0.714 ± 0.014	0.709 ± 0.015	0.775 ± 0.011	–

*The first column corresponds to the performance of individual feature group, hybrid feature, and ensemble learning. The column 2–6 respectively represent the MCC, accuracy, sensitivity, specificity, and AUC value, where each value shown as the average ± SD of 10 alternative balanced datasets. The last column represents a pairwise comparison of AUC between EL and the other methods using a two-tailed *t*-test. *P* ≤ 0.05 indicates a statistically meaningful difference between EL and the selected composition (shown in bold)*.

**Table 3:** Performance comparison of support vector machine (SVM)-based ensemble method with SVM-based other classifiers on benchmarking dataset.

Method	Matthews’ correlation coefficient (MCC)	Accuracy	Sensitivity	Specificity	AUC	*P*-value
Amino acid composition (AAC)	0.219 ± 0.024	0.609 ± 0.012	0.645 ± 0.023	0.573 ± 0.019	0.641 ± 0.016	**0.006**
Dipeptide composition (DPC)	0.269 ± 0.018	0.635 ± 0.009	0.635 ± 0.012	0.634 ± 0.016	0.683 ± 0.009	0.491
Composition–transition–distribution (CTD)	0.182 ± 0.030	0.591 ± 0.015	0.579 ± 0.019	0.603 ± 0.020	0.621 ± 0.016	**0.0003**
Physicochemical properties (PCP)	0.172 ± 0.020	0.585 ± 0.010	0.523 ± 0.035	0.648 ± 0.027	0.620 ± 0.012	**0.0002**
Amino acid index (AAI)	0.228 ± 0.015	0.613 ± 0.007	0.650 ± 0.018	0.577 ± 0.017	0.642 ± 0.010	**0.008**
Hybrid	0.218 ± 0.020	0.609 ± 0.010	0.602 ± 0.014	0.616 ± 0.018	0.647 ± 0.013	**0.015**
Ensemble learning (EL)	0.298 ± 0.022	0.649 ± 0.011	0.618 ± 0.018	0.679 ± 0.009	0.697 ± 0.011	–

*The first column corresponds to the performance of individual feature group, hybrid feature, and ensemble learning. The column 2–6 respectively represent the MCC, accuracy, sensitivity, specificity, and AUC value, where each value shown as the average ± SD of 10 alternative balanced datasets. The last column represents a pairwise comparison of AUC between EL and the other methods using a two-tailed *t*-test. *P* ≤ 0.05 indicates a statistically meaningful difference between EL and the selected composition (shown in bold)*.

Next, we compared the performance PIP-EL with other methods; results are shown in Table 4, where methods are ranked per MCC. This is regarded as one of the best measures in the classification. From Table 4, it is difficult to discriminate the best performance between PIP-EL and ERT, both in terms of accuracy and MCC. However, according to the *P*-value threshold of <0.05, PIP-EL was marginally better than ERT and significantly better than the SVM method, demonstrating that decision tree-based algorithms are more suitable for PIP prediction.

**Table 4:** Performance comparison of PIP-EL with other machine learning-based methods on the same benchmarking dataset.

Method	Matthews’ correlation coefficient (MCC)	Accuracy	Sensitivity	Specificity	AUC	*P*-value
PIP-EL	0.435	0.717	0.701	0.727	0.786	–
Extremely randomized trees (ERT)	0.423	0.712	0.714	0.709	0.775	0.538
Support vector machine (SVM)	0.298	0.649	0.618	0.679	0.697	**<0.000003**
ProInflam	0.580	0.778	0.936	0.620	0.880	–

*The first column represents the methods developed in this study. The column 2–6 respectively represent the MCC, accuracy, sensitivity, specificity, and AUC value. The last column represents a pairwise comparison of AUC between PIP-EL and the other methods using a two-tailed *t*-test. *P* ≤ 0.05 indicates a statistically meaningful difference between PIP-EL and the selected composition (shown in bold). For comparison, we have also included ProInflam CV performance*.

For comparison, we also included ProInflam CV performance, using an imbalanced dataset, reported in Ref. (6). Although it is not intuitive to compare the performance between ProInflam and other methods developed in this study, owing to the variation in the benchmarking dataset, between the sensitivity and specificity [i.e., ΔS = absolute (Sensitivity − Specificity)] between these methods. Here, a smaller value of S is considered more balanced performance. Results show that PIP-EL prediction was more balanced with a ΔS value of 3%, whereas the corresponding value of ProInflam was 26%. This clearly indicates that our approach resulted in balanced performance.

### Effectiveness of Balancing Dataset Approach

**Figure 4:**
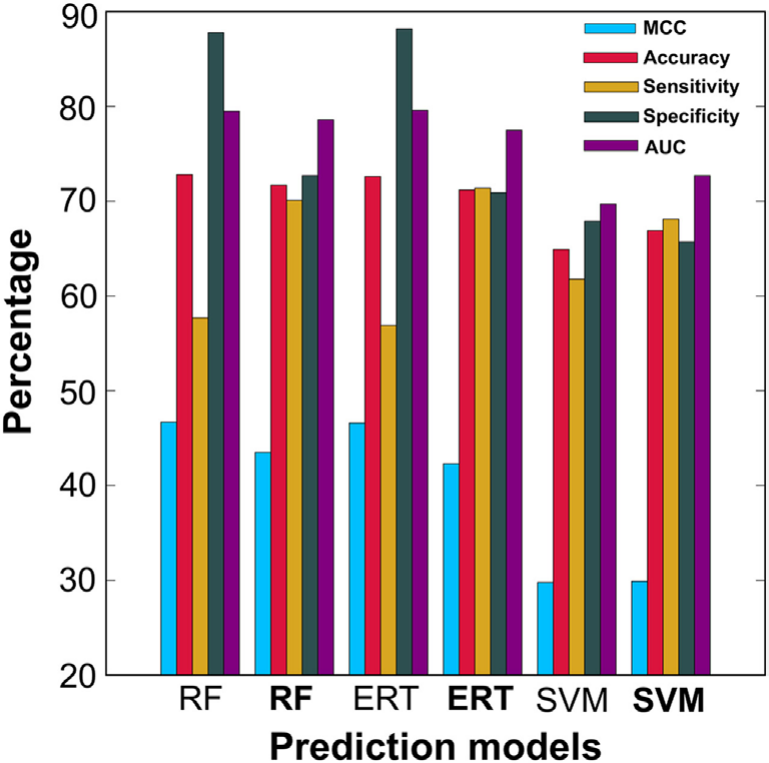
Performance comparison of ensemble models developed using balanced dataset and unbalanced dataset. In the *x*-axis, normal font and bold respectively represent the ensemble model developed using unbalanced and balanced datasets.

In addition to PIP-EL, SVM and ERT, we also generated their corresponding models using an imbalanced dataset. The balanced dataset contained 50 models for each predictor, whereas the imbalanced dataset contained only five models for each predictor. As expected, the performances of the imbalanced dataset-based models were marginally better than the balanced dataset-based models, in terms of MCC and accuracy (Figure 4). However, in terms of more balanced performance (i.e., ΔS), the balanced dataset-based models (i.e., PIP-EL, SVM, and ERT) produced an average ΔS value of 3%, whereas the corresponding metrics in the unbalanced dataset-based models was 32%, indicating that the unbalanced dataset-based models produced biased predictions and misleading accuracies. This analysis clearly shows the importance of handling an imbalanced dataset during prediction model development, regardless of the ML algorithms.

### Evaluation of PIPs Prediction With an Independent Dataset

**Figure 5:**
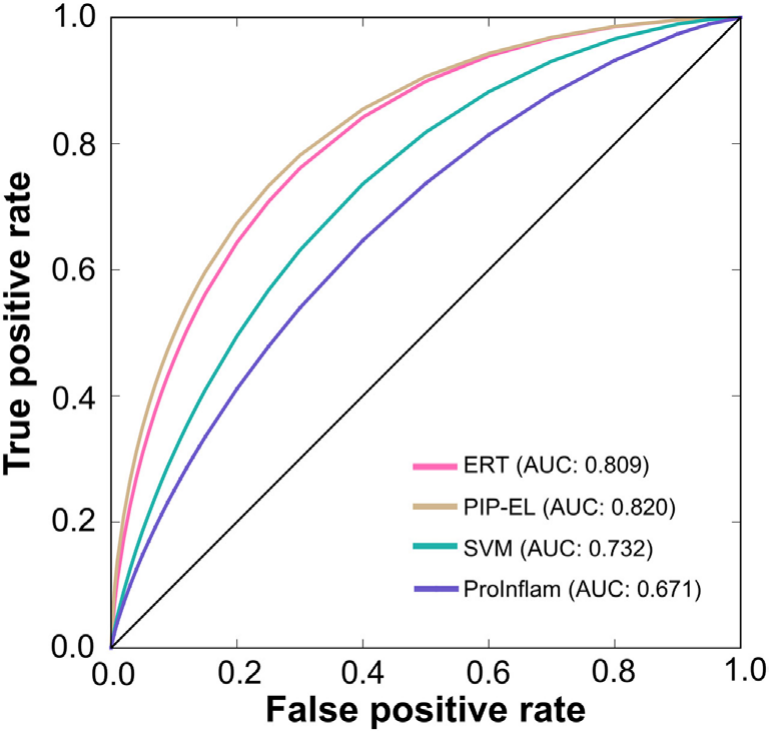
A graphical illustration to show the performance of the method by which receiver operating characteristics curves are obtained from the independent test. *X*- and *Y*-axes, respectively, correspond to the false positive and true positive rates.

To assess the generalization of the models and their ability to perform with unseen data, we evaluated the performances of our three methods with that of the state-of-the-art method (i.e., ProInflam) with an independent dataset. Table 5 shows that PIP-EL achieved values of 0.454 and 0.748 for MCC and accuracy, respectively. Indeed, the corresponding metrics were ~2–35 and ~1–21%, higher than those achieved by other methods, indicating superiority of PIP-EL. Interestingly, PIP-EL performed consistently well, both with benchmarking and on an independent dataset, suggesting its ability to do well with unseen peptides when compared to other ML-based models developed during this study. According to the *P*-value <0.05, PIP-EL performed better than ERT and significantly better than SVM and ProInflam (Figure 5).

**Table 5:** Performance comparison of the PIP-EL with other methods on independent dataset.

Method	Matthews’ correlation coefficient (MCC)	Accuracy	Sensitivity	Specificity	AUC	*P*-value
PIP-EL	0.454	0.748	0.725	0.772	0.820	–
Extremely randomized trees (ERT)	0.433	0.737	0.713	0.762	0.809	0.716
Support vector machine (SVM)	0.332	0.683	0.647	0.720	0.732	**0.006**
ProInflam	0.100	0.537	0.922	0.152	0.671	**0.000007**

*The first column represents the method employed in this study. The column 2–6 respectively represent the MCC, accuracy, sensitivity, specificity, and AUC value. The last column represents a pairwise comparison of AUC between PIP-EL and the other methods using a two-tailed *t*-test. *P* ≤ 0.05 indicates a statistically meaningful difference between PIP-EL and the selected composition (shown in bold)*.

### Web Server Implementation

Establishing free webservers (69–74) or database (75–77) will provide more convenience for most of the wet-experiment scholars. Several instances of bioinformatics web servers utilized for protein function prediction have been reported (3, 78–83) and are of great practical use to the scientific community. Therefore, the online prediction server for PIP-EL was developed.^3^ All datasets used in this study can be downloaded from our web server. PIP-EL represents the second publicly available method for PIP prediction and delivers a higher level of accuracy than ProInflam.

## Discussion

Identifying the epitopes or peptides that induce proinflammatory responses is one of the most challenging tasks of vaccine design; it is of great importance in immunology and peptide therapeutics. The computational identification of PIPs from a given primary sequence remains one of the most challenging problems for immunoinformaticians and computational biologists. In this study, we presented novel software, PIP-EL, which allowed us to predict whether a given peptide induced proinflammatory cytokines, based on the features derived from a set of experimentally validated PIPs and non-PIPs.

First, we constructed an nr dataset of experimentally validated PIPs and non-PIPs extracted from the IEDB, whose size was ~4-fold bigger than the dataset used in the state-of-the-art method (i.e., ProInflam). Interestingly, our nr dataset contained nine proinflammatory cytokines (i.e., IL1α, IL1β, TNFα, IL6, IL8, IL12, IL17, IL18, and IL23), including six used in ProInflam. Compositional and positional preference analyses revealed that Leu and Arg is highly abundant in PIPs, compared to non-PIPs. Previous studies showed that Leu-rich and Arg-rich peptides play an important role in inducing pro-inflammatory cytokines in different autoimmune diseases (84–87) and collage-induced arthritis (88), respectively. Furthermore, determining the biological significance of various dipeptides in proinflammatory induction, observed in our study, requires further studies and experimental validation.

We explored various ML algorithms (i.e., RF, ERT, and SVM) to build models for predicting PIPs. Furthermore, we used a wide range of compositional features for discriminating PIPs and non-PIPs. Note that all five compositions and ML algorithms were used in various sequence-based classification techniques (30, 42, 43, 66, 67, 89–95). However, only two compositions (i.e., AAC and DPC) and SVM were used with previous PIP prediction (6). Generally, ML algorithms produce bias predictions and misleading accuracies when dealing with an imbalanced dataset (63). Although several solutions for the imbalanced problem have been proposed in the literature (63, 64), we chose the most straightforward random under-sampling technique. Finally, an EL approach, called PIP-EL, was developed by fusing an array of 50 RFMs (see Results), which is computationally expensive and has been shown to produce more accurate and robust results, compared to individual composition-based or hybrid models. Although this approach has been successfully applied in various bioinformatics applications (65–67), this is the first instance that this approach has been applied to PIP predictions. Interestingly, PIP-EL performances, both on benchmarking and independent datasets, were more balanced, with an average ΔS of 4%, whose difference is ~9-fold bigger (i.e., 36%) in ProInflam. This is because the authors used an imbalanced dataset for prediction model development, indicating the importance of special handling for the imbalanced dataset during prediction model development.

PIP-EL performed better than the other two methods developed in this study. It performed significantly better than the existing method when objectively evaluated on an independent dataset. The improved performance of PIP-EL was primarily caused by the larger size of benchmarking dataset, random sampling technique followed by EL, rigorous optimisation procedure to select final ML parameters, and the choice of ML method. In future work, it will be beneficial to identify novel contributions that can be used in combination with the current feature set to further improve prediction performance.

## Conclusion

Our proposed method is very promising for PIP prediction. Thus, a user-friendly web interface was made available, allowing researchers access to our prediction method. Although, PIP-EL represents the second publicly available method for predicting PIPs, the delivery of higher accuracy is remarkable. Compared to experimental approaches, bioinformatics methods (e.g., PIP-EL) represent a powerful and cost-effective approach to the proteome-wide prediction of PIPs. Therefore, PIP-EL should be useful for large-scale PIP prediction, facilitating hypothesis-driven experimental design.

## Ethics Statement

The authors declare that there are no ethics problem.

## Author Contributions

Conceived and designed the experiments: BM and GL. Performed the experiments: BM. Analyzed the data: BM and TS. Contributed reagents/materials/software tools: GL and MK. Wrote paper: BM and GL.

## Conflict of Interest Statement

The authors declare that the research was conducted in the absence of any commercial or financial relationships that could be construed as a potential conflict of interest.

## Footnotes

^2^http://www.twosamplelogo.org

^3^www.thegleelab.org/PIP-EL.
