# Supplementary material for: PIP-EL: A New Ensemble Learning Method for Improved Proinflammatory Peptide Predictions
Source: Front Immunol. 2018 Jul 31;9:1783. doi: 10.3389/fimmu.2018.01783 (PMC6079197; doi:10.3389/fimmu.2018.01783)
Supplement: Supplementary file 1 [file Table_1.PDF]

Supplementary Table

Table S1. Hyperparameters optimized by grid search

Parameter	Tested values
Number of estimators (n_estimators[a])	60–1000 with an interval of 20
Maximum number of features considered per split (max_features[a])	1–20 with an interval of 1, "sqrt[b]", None[c]
Minimum number of samples required to split an internal node (min_samples_split[a])	2–10 with an interval of 1

[a] Parameter name in the scikit-learn implementation. [b] default value. [c] all features are used.
